# 1753. Neutropenia as a Function of Ganciclovir Exposure During Treatment of Congenital Cytomegalovirus Disease

**DOI:** 10.1093/ofid/ofad500.1584

**Published:** 2023-11-27

**Authors:** Javier K Nishikawa, Inmaculada Aban, Edward Acosta, Pablo J Sanchez, David Kimberlin

**Affiliations:** University of Alabama Birmingham, Heersink School of Medicine, Huntsville, Alabama; University of Alabama at Birmingham, BIRMINGHAM, Alabama; University of Alabama Birmingham, Birmingham, Alabama; Nationwide Children's Hospital - The Ohio State University, Columbus, OH; University of Alabama at BIrmingham, Birmingham, Alabama

## Abstract

**Background:**

Congenital cytomegalovirus (cCMV) infection is the leading cause of non-genetic sensorineural hearing loss (SNHL) in childhood. Treatment of infants with symptomatic cCMV disease with intravenous (IV) ganciclovir or oral valganciclovir improves audiologic outcomes when started in the first month of age. Neutropenia is the most common adverse event of antiviral treatment, occurring in 19-63% of infants. No correlation between ganciclovir drug exposure and development of neutropenia has been identified.

**Methods:**

We utilized pharmacokinetic (PK), pharmacodynamic (PD), and hematologic data from three international, NIH-funded studies of IV ganciclovir or oral valganciclovir conducted by the Collaborative Antiviral Study Group (CASG) from 2002-2018. The minimum absolute neutrophil count (ANC) was used to classify each patient as life-threateningly, severely, moderately, mildly or never neutropenic, as defined in the Division of AIDS Toxicity Tables. The mean 12-hour area under the curve (AUC_12_) values of patients from each category were compared using ANOVA. The correlation between the minimum ANC value and the AUC value was estimated using Pearson correlation. Ordinal logistic regression determined if AUC was a significant predictor of neutropenia category.

**Results:**

139 subjects were included, with 18 (13%) developing an ANC below 500 prompting suspension of antiviral treatment. AUC_12_ exposure as a function of degree of neutropenia is presented in Figure 1. AUC_12_ weakly correlated with the ANC (p=0.02; R=0.20), suggesting that higher AUC values very weakly correlated with higher ANC values, the opposite of what was hypothesized. In comparison, the ANOVA showed no significant difference (P=0.72) between each group of subjects, meaning that each category of neutropenia status had equivalent drug exposure. Ordinal logistic regression did not show that AUC_12_ is a significant predictor of neutropenia category (P=0.62).

**Figure 1:**
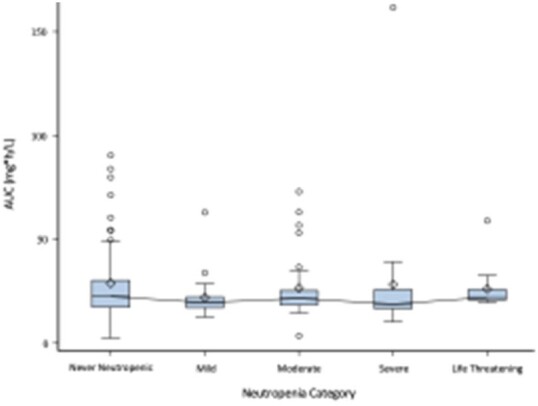
AUC Values Across Five Categories of Neutropenia

**Conclusion:**

We did not observe a high correlation between AUC_12_ and neutropenia. However, most participants only had one AUC_12_ result from time of study enrollment, so our results do not exclude the possibility that such a correlation exists. Future analyses will explore additional PK parameters, such as C_max_ and half-life, as a function of ANC values.

**Disclosures:**

**Inmaculada Aban, PHD**, Roche: Steering Committee Member **David Kimberlin, MD**, Gilead: Grant/Research Support|Gilead: Served as site PI on pediatric remdesivir study. All monies went directly to my university and not to me.

